# Extracellular vesicles secreted by human gingival mesenchymal stem cells promote bone regeneration in rat femoral bone defects

**DOI:** 10.3389/fbioe.2023.1098172

**Published:** 2023-02-21

**Authors:** Situo Wang, Ziwei Liu, Shuo Yang, Na Huo, Bo Qiao, Tong Zhang, Juan Xu, Quan Shi

**Affiliations:** ^1^ Department of Stomatology, The First Medical Center, Chinese PLA General Hospital, Beijing, China; ^2^ Orthopedic Laboratory of PLA General Hospital, Beijing, China; ^3^ Medical School of Chinese PLA, Beijing, China

**Keywords:** extracellular vesicles, human gingival mesenchymal stem cells, osteogenic ability, angiogenic capability, bone regeneration

## Abstract

Extracellular vesicles (EVs), important components of paracrine secretion, are involved in various pathological and physiological processes of the body. In this study, we researched the benefits of EVs secreted by human gingival mesenchymal stem cells (hGMSC-derived EVs) in promoting bone regeneration, thereby providing new ideas for EVs-based bone regeneration therapy. Here, we successfully demonstrated that hGMSC-derived EVs could enhance the osteogenic ability of rat bone marrow mesenchymal stem cells and the angiogenic capability of human umbilical vein endothelial cells. Then, femoral defect rat models were created and treated with phosphate-buffered saline, nanohydroxyapatite/collagen (nHAC), a grouping of nHAC/hGMSCs, and a grouping of nHAC/EVs. The results of our study indicated that the combination of hGMSC-derived EVs and nHAC materials could significantly promote new bone formation and neovascularization with a similar effect to that of the nHAC/hGMSCs group. Our outcomes provide new messages on the role of hGMSC-derived EVs in tissue engineering, which exhibit great potential in bone regeneration treatment.

## 1 Introduction

The repair and regeneration of bone defects caused by tumors, trauma, and infection have always been a hot issue in the field of orthopedics and stomatology (Nguyen, et al., 2018). Autologous bone grafts are considered the “gold standard” for bone repair (Kumar, et al., 2016). However, they have some disadvantages, such as the need for a secondary operation, defects in the donor site, and unpredictable autogenous bone absorption (Valtanen, et al., 2021).

In recent years, tissue engineering strategies based on mesenchymal stem cells (MSCs) have been widely used in the field of bone regeneration. Human gingival mesenchymal stem cells (hGMSCs) are adult stem cells isolated from the gingival lamina propria with multidirectional differentiation potential and high proliferation characteristics; in addition, they have abundant sources and can be easily harvested minimal invasively (Fawzy El-Sayed and Dörfer, 2016). Compared with bone marrow mesenchymal stem cells (BMSCs), hGMSCs have the advantages of faster proliferation and more stable morphology *in vitro* (Tomar, et al., 2010). In addition, as the majority of hGMSCs are derived from cranial neural crest cells, hGMSCs have good tissue regeneration and immunomodulation functions (Xu, et al., 2013). hGMSCs have been extensively investigated for bone regeneration and have shown good application effects (Al-Qadhi, et al., 2020; Hasani-Sadrabadi, et al., 2020). However, there are disadvantages to MSC transplantation, such as a low survival rate of transplanted cells, tumorigenic effects and immunological rejection (Eggenhofer, et al., 2014). Therefore, avoiding the risk of using MSCs or finding substitutes for MSCs to achieve cell-free therapy is one of the problems to be solved at present.

A recent basic study indicated that the tissue repair function of MSCs is mainly exerted by paracrine secretion of bioactive molecules (Liang, et al., 2014; Najar, et al., 2021; Williams et al., 2022). As an important paracrine factor, extracellular vesicles (EVs) are lipid bilayer nanovesicles secreted by living cells and their classification and nomenclature were formulated by the International Society of Extracellular Vesicles (ISEV) (Boere, et al., 2018). EVs carry a variety of bioactive molecules, such as microRNAs (miRNAs), mRNAs, lipids, and proteins, and are widely distributed in body fluids, such as breast milk, saliva, urine and bile (Trajkovic, et al., 2008; Rani, et al., 2015). After being secreted, EVs can be absorbed by receptor cells through ligand/receptor recognition, membrane fusion or phagocytosis and can regulate cell-to-cell communication by transmitting bioactive molecules (Tarasov, et al., 2021). It has been reported that MSC-derived EVs have shown remarkable therapeutic effects in many disease models, such as cardiovascular diseases, nervous system diseases and immune system diseases (Moghadasi, et al., 2021). Accumulating studies have shown that MSC-derived EVs can effectively promote the repair and regeneration of bone defects, and this effect is closely related to the regulation of osteogenesis and angiogenesis-related cells by MSC-derived EVs (Qin, et al., 2016). However, the therapeutic effect of hGMSC-derived EVs on bone defect repair and regeneration is unclear.

Therefore, in this study, we examined the effect of hGMSC-derived EVs on osteogenesis and angiogenesis by treating rat bone marrow mesenchymal stem cells (rBMSCs) and human umbilical vein endothelial cells (HUVECs) with hGMSC-derived EVs. In addition, we evaluated the bone repair capacity of nanohydroxyapatite/collagen (nHAC) scaffolds loaded with hGMSC-derived EVs on rat femoral defects. Our study showed that the combination of hGMSC-derived EVs/nHAC could promote the repair and regeneration of bone defects by accelerating new bone formation and angiogenesis, potentially providing application value for the treatment of bone defects.

## 2 Materials and methods

### 2.1 Cell isolation and culture

The methods for extraction and culture of primary hGMSCs were as previously described (Shi, et al., 2017). Gingival tissue was obtained from healthy young patients undergoing tooth crown lengthening operation, impacted third molar extraction and secondary implant surgery. Briefly, the gingival tissue was digested in 2 mg/ml dispase (Roche) at 4°C for 12 h after several rinses with phosphate-buffered saline (PBS). Then, the lamina propria was separated from the gingival tissue, minced and digested with 2 mg/ml collagenase IV (Roche) at 37 °C for 1 h. Afterward, the cell and tissue pellets were cultured in Dulbecco’s modified Eagle’s medium, nutrient mixture F-12 (DMEM/F12, Gibco) supplemented with 10% fetal bovine serum (FBS, Gibco), 100 U/ml penicillin, 100 μg/ml streptomycin and 0.25 μg/ml amphotericin B at 37°C with 5% CO2. hGMSCs at passages three to six were used in this experiment. The methods for extraction and culture of primary rBMSCs were as previously described (Liu, et al., 2020). Briefly, bone marrow was flushed from the femoral bones of SD suckling rats using α-minimum essential medium (α-MEM, Gibco) supplemented with 10% fetal bovine serum (FBS, Gibco), 100 U/ml penicillin, 100 μg/ml streptomycin and 0.25 μg/ml amphotericin B. The cell and tissue pellets were then cultured in α-MEM complete medium. HUVECs were purchased from PROCELL (Wuhan, China) and cultured in HUVEC special medium (Procell). All experimental procedures obtained approval from Clinical Ethics Committee of the Chinese PLA General Hospital.

### 2.2 Isolation and identification of hGMSC-Derived EVs

hGMSCs were cultured in exosome-free FBS medium to collect conditioned medium (Théry, et al., 2006). First, the cells were cultured in osteogenic induction medium (OM):α-MEM supplemented with 10% FBS, 0.1 μmol/L dexamethasone (Gibco), 10 mmol/L β-glycerol sodium phosphate (Gibco) and 50 μg/mL ascorbic acid (Gibco) for 3 days, then OM was replaced with α-MEM medium supplemented with 10% exosome-free FBS for culture with an additional 2 days to collect the conditioned medium. Afterward, the conditioned medium was centrifuged at 500 × g for 10 min and 1,000 × g for 30 min and then filtered through a 0.22 μm sterilized filter. The filtered medium was added to an ultrafiltration centrifuge tube (15 ml Amicon Ultra 30kD, Millipore) and centrifuged at 5000 × g for 20 min to concentrate the medium. Subsequently, the concentrated supernatant was ultracentrifuged at 100,000 × g for 60 min, and then the supernatant was replaced with PBS for the same operation for 60 min to obtain the EVs. The EVs were stored at −80°C.

The morphology of EVs was observed by transmission electron microscopy (TEM, HITACHI). Briefly, 5 μl of EVs was loaded onto a copper grid for 5 min, and the excess liquid was removed by filter paper. After staining with 2% uranyl acetate dihydrate for 1 min, the sample was detected by TEM. The particle size distribution was examined by using nanoparticle tracking analysis (NTA). In addition, Western blotting was performed according to standard protocol as previously reported (Swanson, et al., 2020) to detect the EVs marker CD9 (ab236630, Abcam), tumor susceptibility gene (Tsg) 101 (ab133586, Abcam) and heat shock protein (Hsp) 70 (ab5439, Abcam). All antibodies were diluted at a concentration ratio of 1:1,000. The protein concentrations of the EVs were measured by using the BCA Protein Assay Kit (Servicebio).

### 2.3 BMSC osteogenic differentiation assay

Four groups were established as follows: 1) OM (control), 2) OM complemented with 25 μg/ml hGMSC-derived EVs (25 μg/ml EVs), 3) OM complemented with 50 μg/ml hGMSC-derived EVs (50 μg/ml EVs), and 4) OM complemented with 100 μg/ml hGMSC-derived EVs (100 μg/ml EVs).

Alkaline phosphatase (ALP) staining (Beyotime) and an ALP assay kit (Beyotime) were used to assess ALP activity after 14 days of osteoinduction. Alizarin red staining (Solarbio) was conducted to assess mineralization following 14 days of osteoinduction. To quantify the matrix calcifications, the calcium was deposited with 10% cetylpyridinium chloride (Sigma) for 60 min and measured by the absorbance at 562 nm.

To further examine the expression of osteogenesis-related genes and proteins, real-time qPCR and Western blotting were conducted. The operation steps of real-time qPCR are briefly described as follows. Total RNA was extracted from cells by TRIzol (Servicebio) after 14 days of osteoinduction and then synthesized into cDNA by using StarScript III RT Mix (Genstar). Afterward, quantitative polymerase chain reaction was performed using StarScript III SYBR Mix (Genstar). The primers for *ALP*, osteocalcin (*OCN*) and runt-related transcription factor 2 (*RUNX2*) are presented in Table 1. In addition, total protein was isolated from cells using cell lysis buffer (Beyotime) after 14 days of osteoinduction. Western blotting was performed to detect the expression of osteogenesis-related proteins ALP (No. 60294-1-Ig, Proteintech), OCN (GTX64348, GeneTex) and RUNX2 (No. 20700-1-AP, Proteintech).

**TABLE 1:** Primers Used for Real-Time qPCR.

Gene	Forward (5′-3′)	Reverse (3′-5′)
*ALP*	TGG​TAC​TCG​GAC​AAT​GAG​ATG​C	GCT​CTT​CCA​AAT​GCT​GAT​GAG​GT
*OCN*	AGG​GCA​GTA​AGG​TGG​TGA​ATA​GA	GAA​GCC​AAT​GTG​GTC​CGC​TA
*RUNX2*	CAG​TAT​GAG​AGT​AGG​TGT​CCC​GC	AAG​AGG​GGT​AAG​ACT​GGT​CAT​AGG
*GAPDH*	GGC​ACA​GTC​AAG​GCT​GAG​AAT​G	ATG​GTG​GTG​AAG​ACG​CCA​GTA

### 2.4 HUVEC angiogenic differentiation assay

HUVECs were cultured in HUVEC special medium with or without different concentrations of EVs (25 μg/ml, 50 μg/ml, and 100 μg/ml). Tube formation assays were performed to assess the impact of hGMSC-derived EVs on angiogenesis. HUVECs pretreated with or without EVs were seeded into 24-well plates covered with Matrigel (BD Biosciences). Images of tube formation were obtained by microscopy after 6 h of culture. ImageJ software was used to quantitatively analyze the number and total length of tubes.

### 2.5 Animal experiment design

#### 2.5.1 Preparation and characterization of nHAC-containing cells and EVs

nHAC materials with diameters of 3.5 mm, comprising collagen I and nanohydroxyapatite, were purchased from Allgens Medical Co., Ltd. (Beijing, China). Small pieces 4 mm in length were cut from the nHAC material with a scalpel as scaffolds. Each scaffold was injected with 50 μl of 4 × 106 cells/ml cell solution and then transferred to 24-well plates.

nHAC materials with hGMSCs were cultured in DMEM/F12 complete medium for 3 days. Scaffolds with or without hGMSCs were observed under a scanning electron microscope (JEOL).

Then, 100 μl of EVs at a concentration of 1 μg/μl was injected into each scaffold. To investigate the loading of EVs in the scaffold, EVs were labeled green with DIO (green) dye (Abmole) according to the manufacturer’s protocol. The control group was injected with the same volume of PBS. Fluorescence expression was examined under a laser scanning confocal microscope (LSCM, ZEISS).

#### 2.5.2 Critical-sized femoral defect model

The animal experiments in this study were approved by Animal Care and Use Committee of Chinese PLA General Hospital. A total of 60 male Sprague-Dawley rats (12 weeks old, SPF) were purchased from Sibeifu Biotechnology Co., Ltd. (Beijing, China). The rats were randomly divided into four groups as follows: 1) defects with PBS treatment (control, *n* = 15); 2) defects treated with nHAC scaffolds (nHAC, *n* = 15); 3) defects treated with nHAC scaffolds loaded with hGMSCs (nHAC/hGMSCs, *n* = 15); and 4) defects treated with nHAC scaffolds loaded with EVs (nHAC/EVs, *n* = 15). The femoral defect model was established as previously described (Wang, et al., 2020). Briefly, the rats were anesthetized by intraperitoneal injection of 2% sodium pentobarbital solution (45 mg/kg). Then, Critical-sized defects of 4 × 4 × 4 mm^3^ were created at the lateral femoral condyle. After 4, 8, and 12 weeks, five animals were sacrificed in each group. Then, the femoral condyle defect sites were obtained and fixed in 4% paraformaldehyde for 48 h.

#### 2.5.3 Gross observation and imaging examination

The specimens were examined under a stereomicroscope (Nikon). X-ray images were then obtained by a Faxitron cabinet X-ray system to observe defect healing. The femoral condyles with defects were scanned with a micro-CT scanner (Skyscan). Three-dimensional (3D) reconstruction was performed and analyzed using 3D visualization software (Skyscan). The BMD, bone volume/tissue volume (BV/TV%), trabecular thickness (Tb. Th), and trabecular separation/spacing (Tb.Sp) were calculated.

#### 2.5.4 Histological and immunohistochemical (IHC) analysis

After micro-CT analysis, the specimens were decalcified using 10% EDTA (pH 7.4) for 30 days, dehydrated and embedded in paraffin. Ultimately, the specimens were cut into 4-μm-thick sections. HE, Masson and Goldner staining were conducted to assess bone healing in the defect sites. To further assess new bone formation and neovascularization in femoral condyle defect sites, immunohistochemical staining for osteogenesis-related protein OCN and angiogenesis-related protein CD34 was performed. The primary antibodies anti-OCN (Servicebio) and anti-CD34 (Servicebio) were diluted 1:500 and used according to the manufacturer’s instructions.

### 2.6 Statistical analysis

All data are presented as the mean ± standard deviation for three experiments per group. Student’s t-test was used for two-group comparisons, and one-way ANOVA was used for comparisons among three or four groups. *p* < 0.05 was considered statistically significant.

## 3 Results

### 3.1 Characterization of hGMSC-derived EVs

**FIGURE 1:**
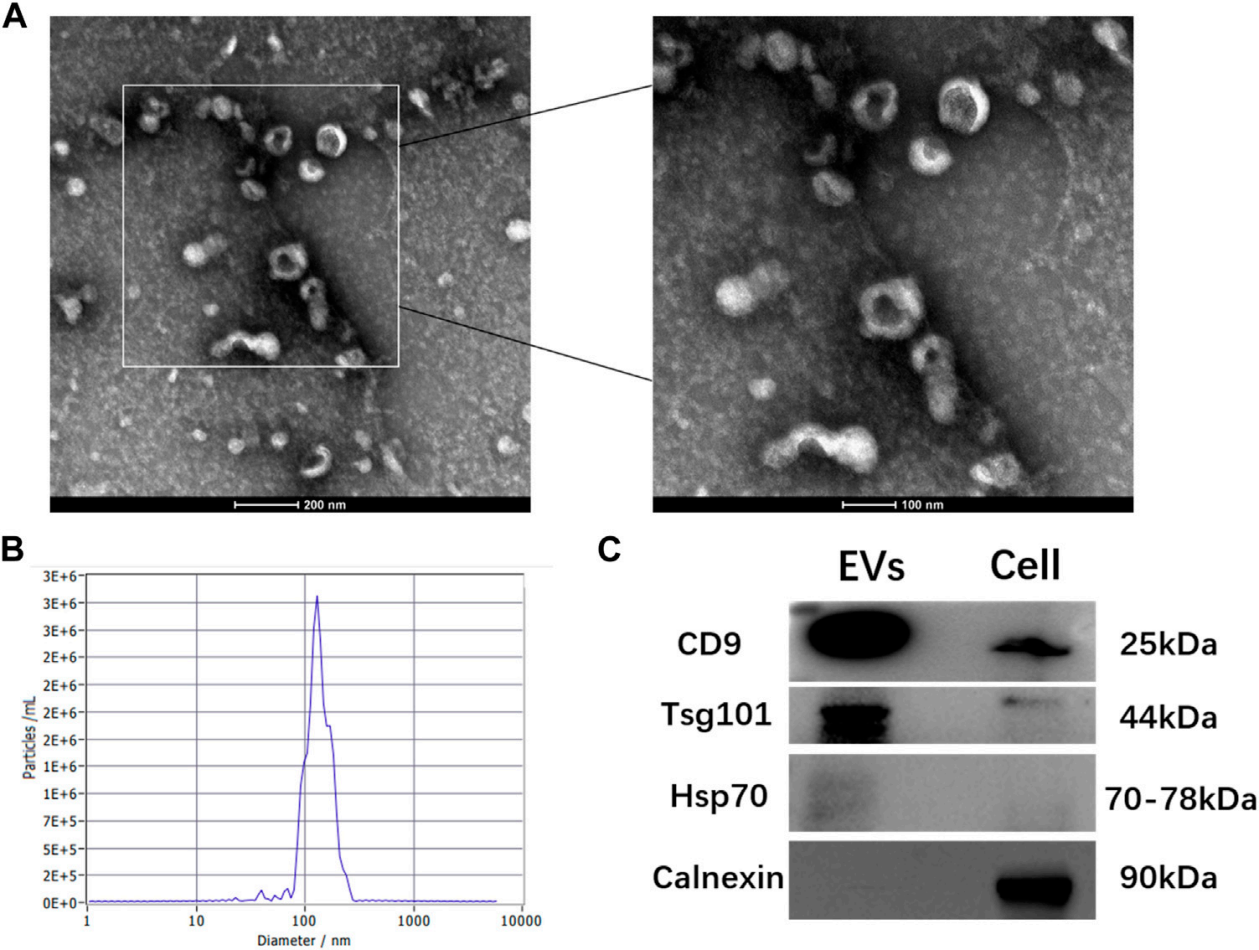
Characterization of hGMSC-derived EVs **(A)** hGMSC-derived EVs morphology observed by TEM **(B)** Particle size distribution of hGMSC-derived EVs detected by NTA **(C)** Western blotting results of the EVs surface markers CD9, Tsg101, and Hsp 70.

The TEM analysis showed that hGMSC-derived EVs had a cup-shaped morphology with a bilayer membrane structure (Figure 1A). The NTA analysis revealed that the peak of the diameter distribution of these nanoparticles was approximately 120 nm, and it was 127.1 ± 37.6 nm in the quantitative analysis (Figure 1B). The Western blotting results demonstrated that hGMSC-derived EVs expressed CD9, TSG101 and HSP70 (Figure 1C).

### 3.2 hGMSC-derived EVs promote the osteogenic differentiation of BMSC

**FIGURE 2:**
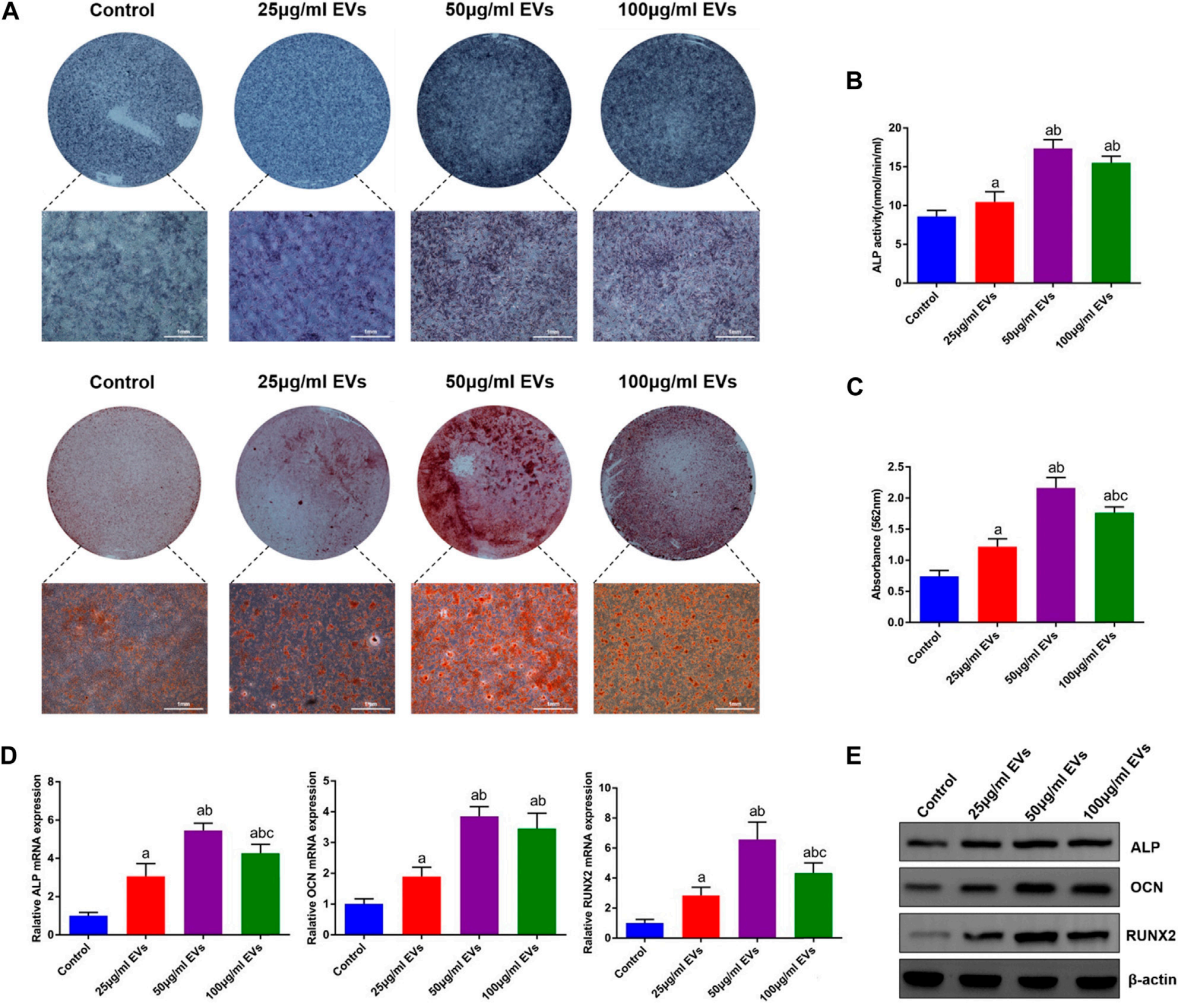
Effects of EVs on the osteogenic differentiation of rBMSCs **(A)** The observation of ALP and alizarin red staining **(B)** Quantification of ALP activity **(C)** Quantification of alizarin red staining **(D)** The expression of osteogenic genes (ALP, OCN and RUNX2) **(E)** The expression of osteogenic proteins (ALP, OCN and RUNX2). a, *p* < 0.05 compared with the control group; b, *p* < 0.05 compared with the 25 μg/ml EVs group; c, *p* < 0.05 compared with the 50 μg/ml EVs group.

To investigate the effect of hGMSC-derived EVs on the osteogenic differentiation of rBMSCs, rBMSCs were cultured in OM with or without different concentrations of EVs (25 μg/ml, 50 μg/ml, and 100 μg/ml). Following 14 days of induction, ALP staining and ALP activity in rBMSCs were significantly increased in the EVs groups compared with the control group, among which the 50 μg/ml EVs group had the best effect (Figures 2A, B). Additionally, alizarin red staining revealed that the mineralization capacity of rBMSCs was enhanced by EVs with the best effect of 50 μg/ml (Figures 2A, C). Likewise, osteogenic mRNA and protein expression (ALP, OCN and RUNX2) was upregulated by EVs, with the highest level in the 50 μg/ml EVs group (Figures 2D, E).

### 3.3 hGMSC-derived EVs promote the angiogenic capacity of HUVEC

**FIGURE 3:**
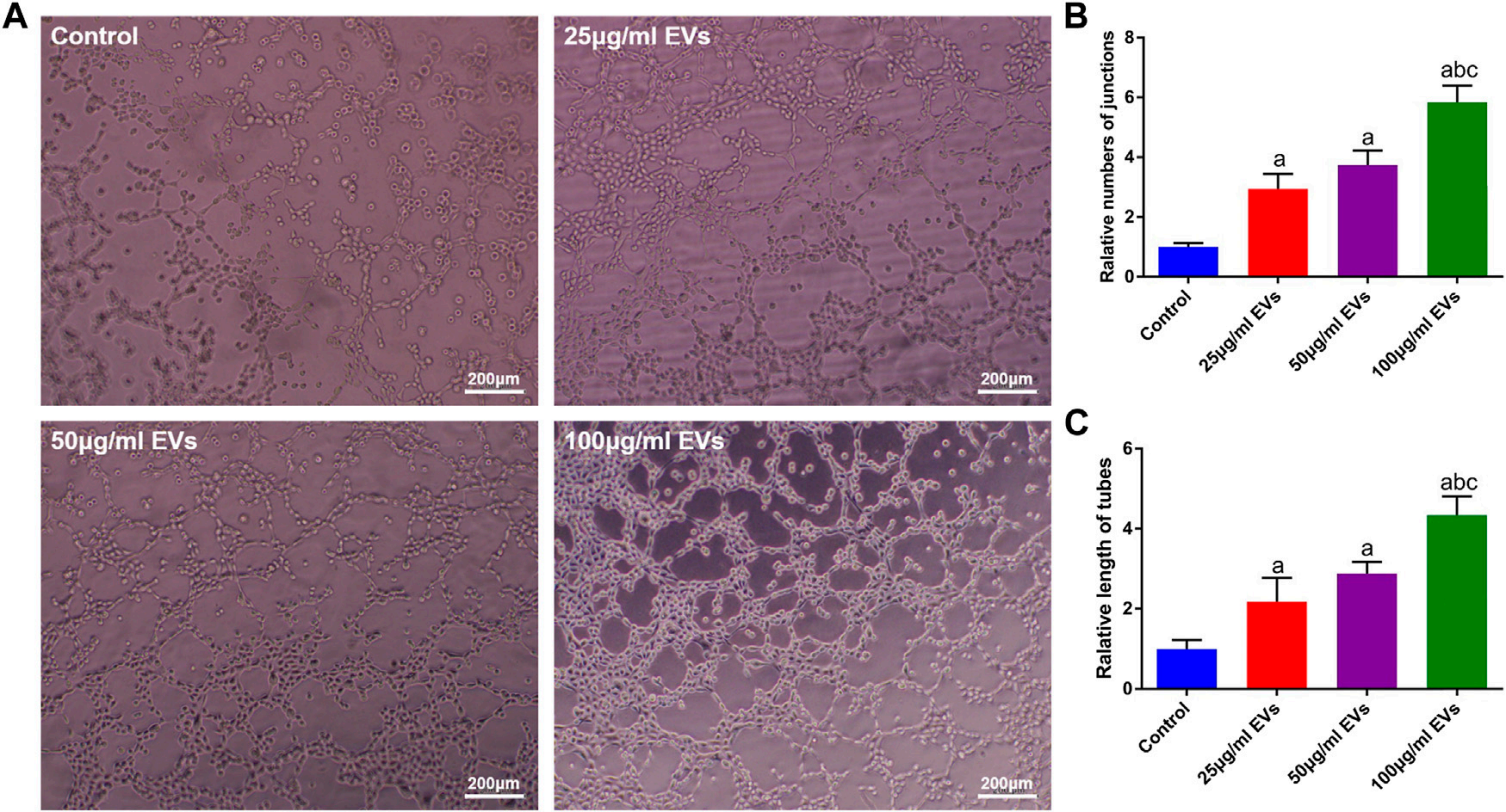
Effects of EVs on the angiogenic capacity of HUVECs **(A)** Image of the tube formation assay **(B)** Quantification analysis of tube numbers **(C)** Quantification analysis of tube length. a, *p* < 0.05 compared with the control group; b, *p* < 0.05 compared with the 25 μg/ml EVs group; c, *p* < 0.05 compared with the 50 μg/ml EVs group.

To evaluate the effect of hGMSC-derived EVs on the angiogenic ability of HUVECs, a tube formation assay was conducted. As shown in Figure 3A, the HUVECs in the EVs groups exhibited stronger angiogenic ability than those in the control group, and this ability was enhanced with increasing EVs concentration. Similarly, the quantitative analysis also showed that the number and total length of tubes were significantly higher in the EVs groups (Figures 3B, C).

### 3.4 hGMSC and EVs detection from the nHAC scaffold

**FIGURE 4:**
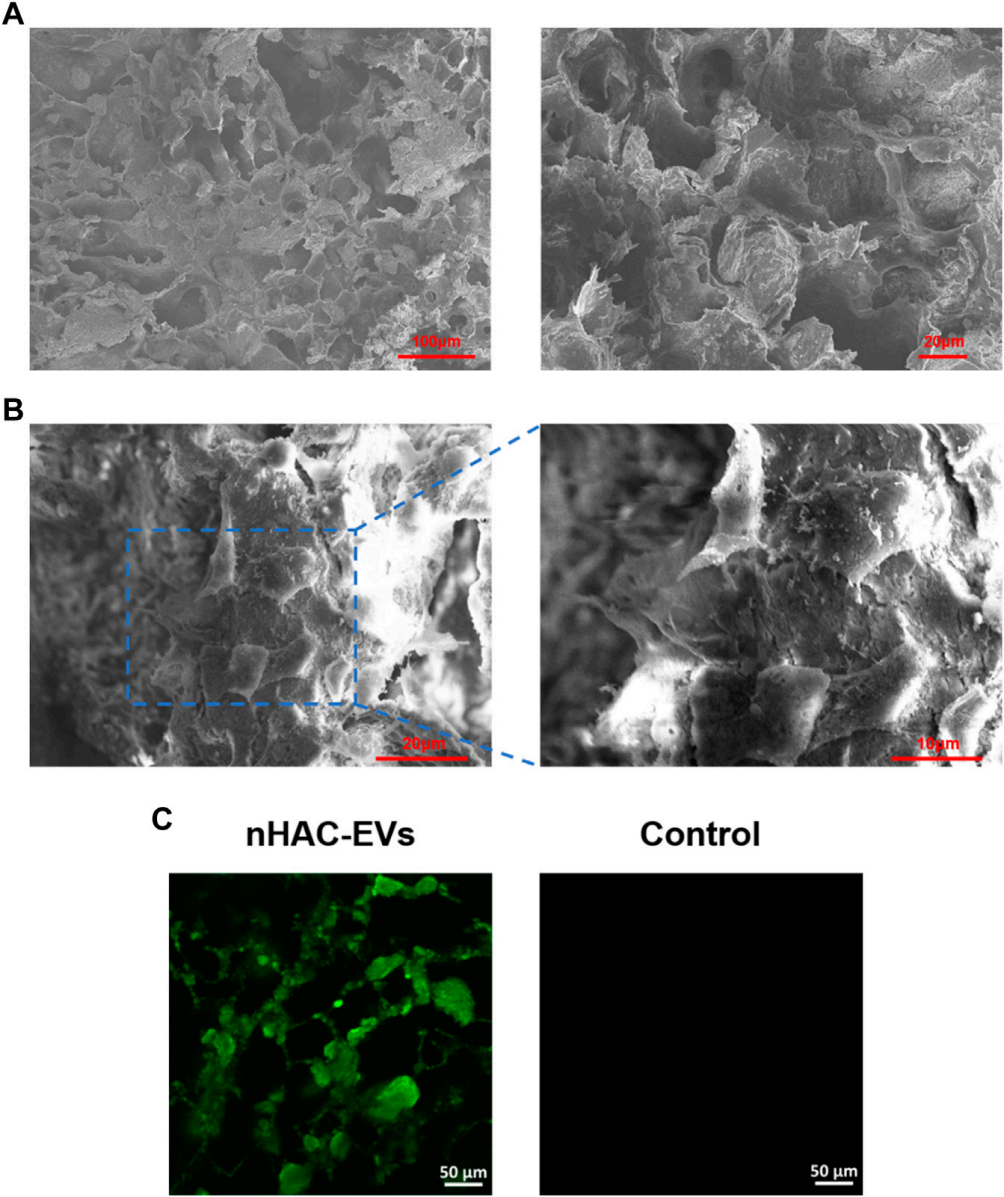
Detection of hGMSCs and EVs on the nHAC scaffold **(A)** SEM image of the nHAC scaffold (a: 200×; b: 600×) **(B)** SEM image of the nHAC scaffold with hGMSCs (a: 1100×; b: 2200×) **(C)** LSCM images of the nHAC scaffold with DiO-labeled EVs.

SEM showed that the nHAC material had a porous structure with a uniform pore size of approximately 50–150 μm (Figure 4A). After 3 days of culture, hGMSCs on the nHAC scaffold grew well and adhered to the surface of the material. Moreover, secreted filamentous extracellular matrix around cells could be observed under high magnification (Figure 4B). After DIO-labeled EVs were added to the nHAC material, a large amount of green fluorescence was observed on the material by LSCM, and no fluorescence was detected in the control group (Figure 4C).

### 3.5 Cross observation and imaging analysis of bone regeneration

**FIGURE 5:**
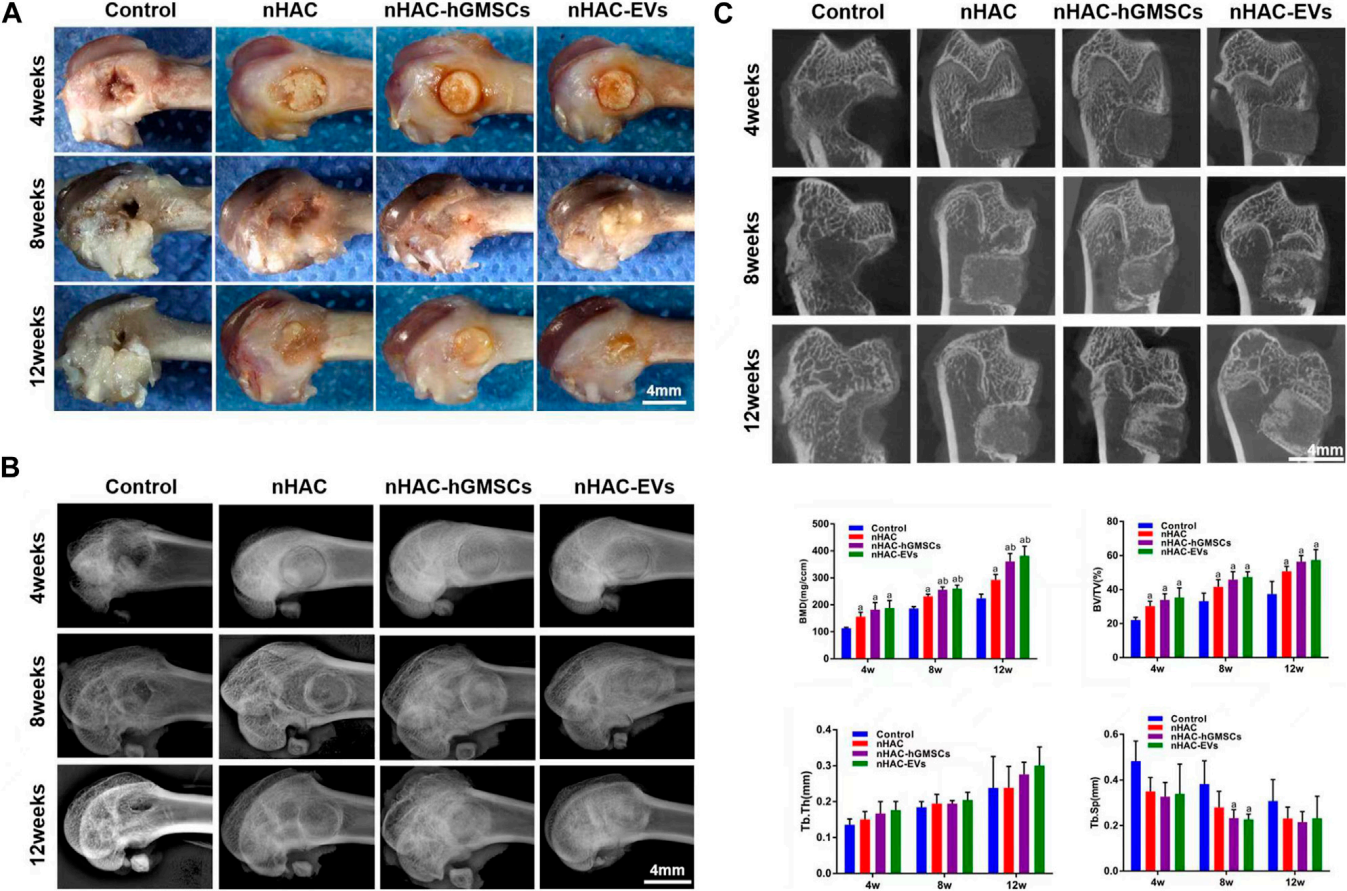
Cross observation and imaging analysis of bone regeneration **(A)** Cross observation images of bone defect sites **(B)** X-ray images of bone defect sites **(C)** Micro-CT images and analysis of bone formation using BMD, BV/TV%, Tb.Th, and Tb. Sp. a, *p* < 0.05 compared with the control group; b, *p* < 0.05 compared with the nHAC group.

A total of 60 Sprague-Dawley rats with femoral defects were divided into four groups (control, nHAC, nHAC/hGMSCs and nHAC/EVs; *n* = 15/group) and euthanized by dislocation of cervical vertebrae under deep anesthesia in three different times (at 4, 8, and 12 weeks). As shown in Figure 5A, defects gradually decreased with healing time, as observed under a stereomicroscope. As expected, the defects of each group implanted with nHAC healed better than those of the control group. Among them, the defect healing of the nHAC/hGMSCs group and nHAC/EVs group was better than that of the nHAC group. Moreover, the results of X-ray and micro-CT imaging also showed that the bone healing effect of the nHAC/hGMSCs group and nHAC/EVs group was better than that of the other two groups, and the defects in the nHAC group healed better (Figures 5B, C). According to 3D reconstruction analysis, the BMD, BV/TV%, Tb.Th, and Tb. Sp results further revealed that more new bone formation was discovered in the HAC/hGMSCs group and nHAC/EVs group than in the nHAC group and control group. In addition, no significant difference was found in the amount of new bone formation between the HAC/hGMSCs group and the nHAC/EVs group (Figure 5C).

### 3.6 Histological results of bone regeneration

**FIGURE 6:**
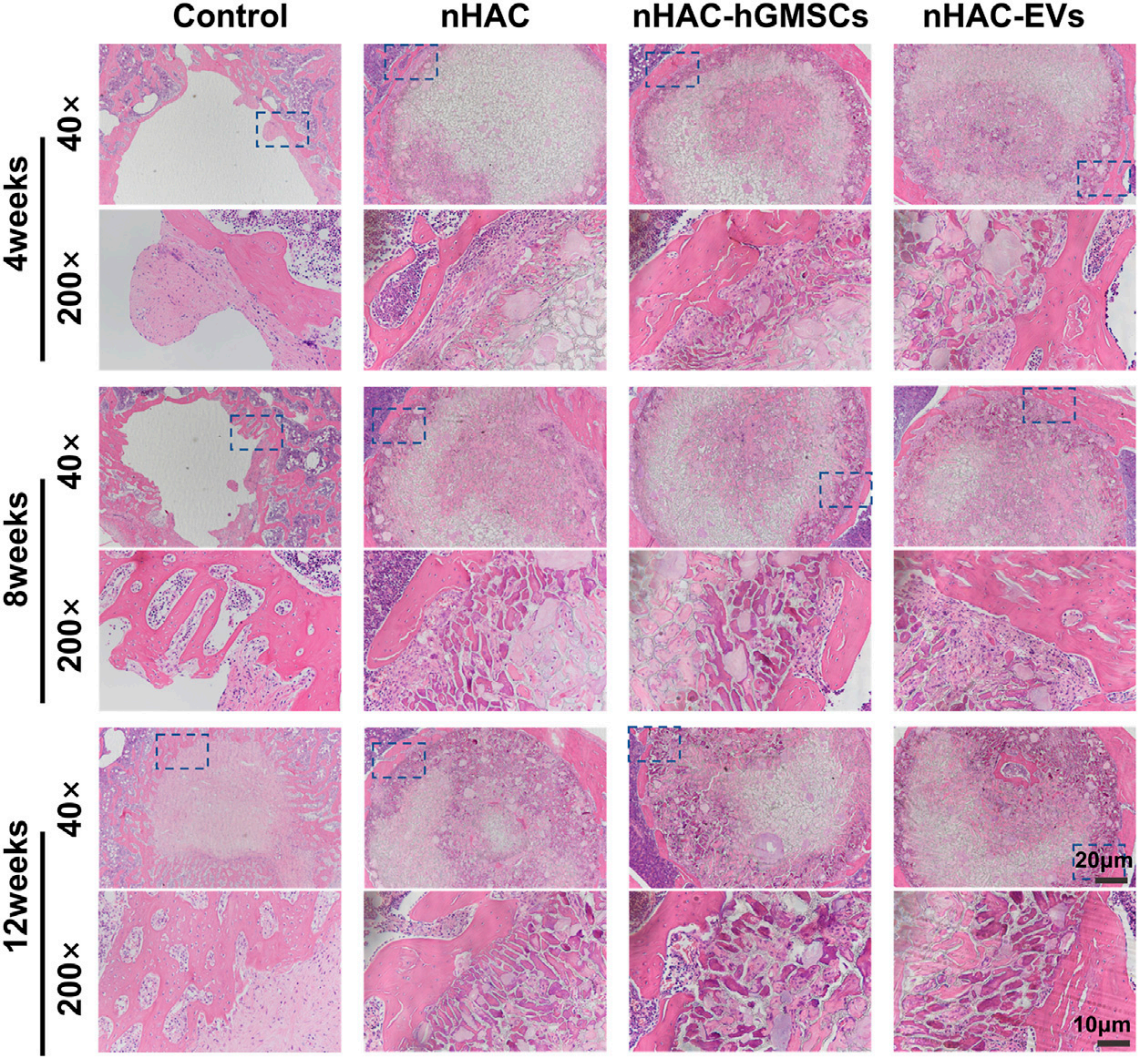
Representative images of HE staining of the bone defect area. The 200× image is an enlargement in the dashed box of the 40× image.

**FIGURE 7:**
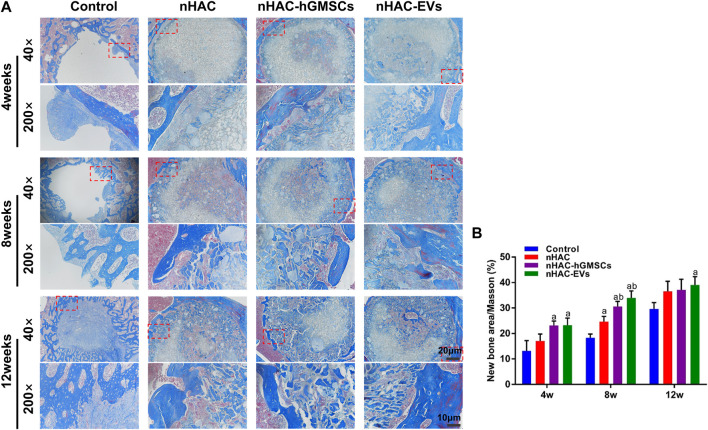
Masson staining results of bone regeneration **(A)** Representative images of Masson staining of the bone defect area. The 200× image is an enlargement in the dashed box of the 40× image **(B)** Quantitative analysis of new bone. a, *p* < 0.05 compared with the control group; b, *p* < 0.05 compared with the nHAC group.

**FIGURE 8:**
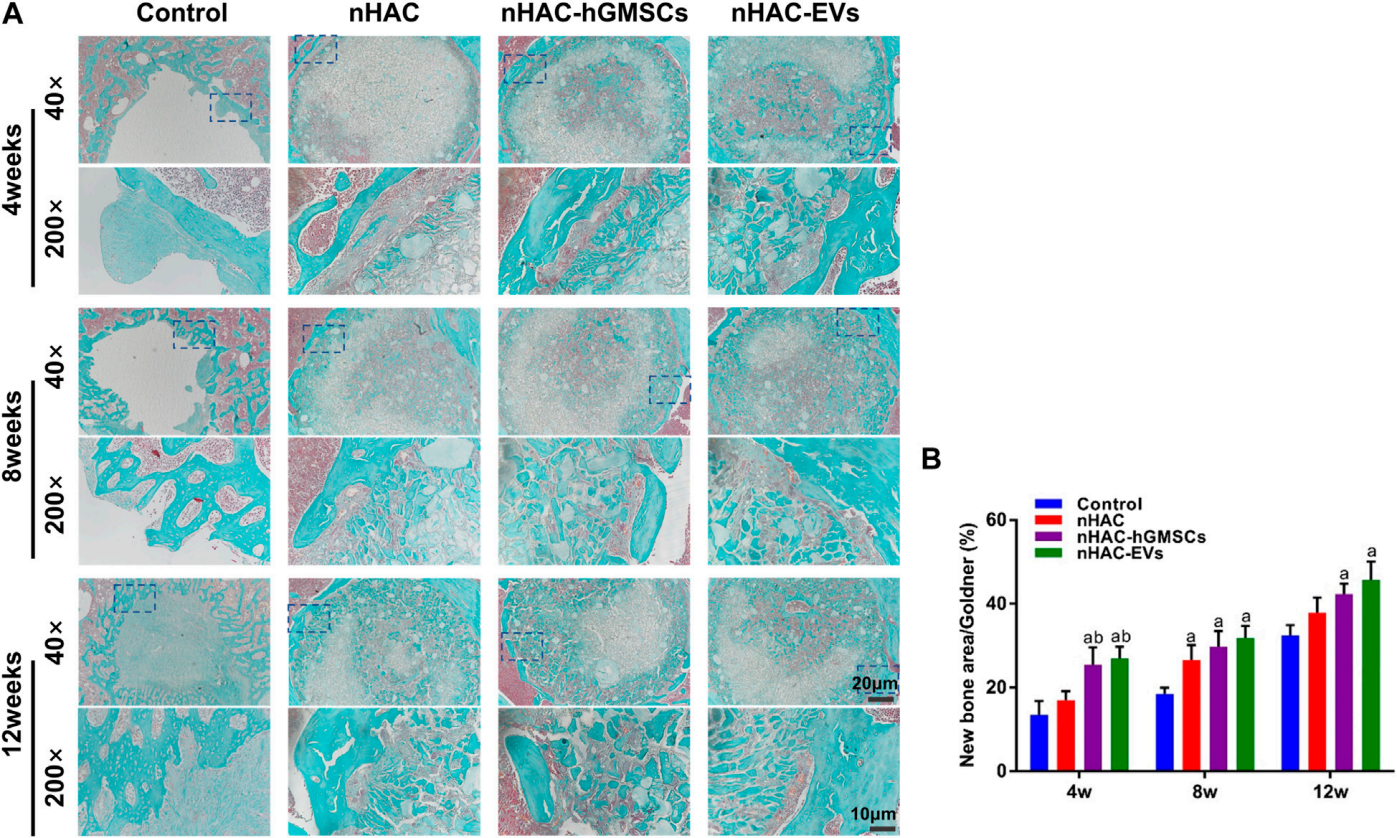
Goldner staining results of bone regeneration **(A)** Representative images of Goldner staining of the bone defect area. The 200× image is an enlargement in the dashed box of the 40× image **(B)** Quantitative analysis of new bone. a, *p* < 0.05 compared with the control group; b, *p* < 0.05 compared with the nHAC group.

The HE staining results at 4, 8, and 12 weeks are shown in Figure 6. The regenerative bone mass in the femoral defect area increased over time in each group, although it was higher in groups implanted with nHAC, among which the nHAC/hGMSCs group and nHAC/EVs group were better. The Masson and Goldner staining results indicated that the nHAC/hGMSCs group and nHAC/EVs group at each time point had more collagen deposition and new bone formation than the other two groups (Figures 7, 8).

### 3.7 Immunohistochemical staining results of bone regeneration

**FIGURE 9:**
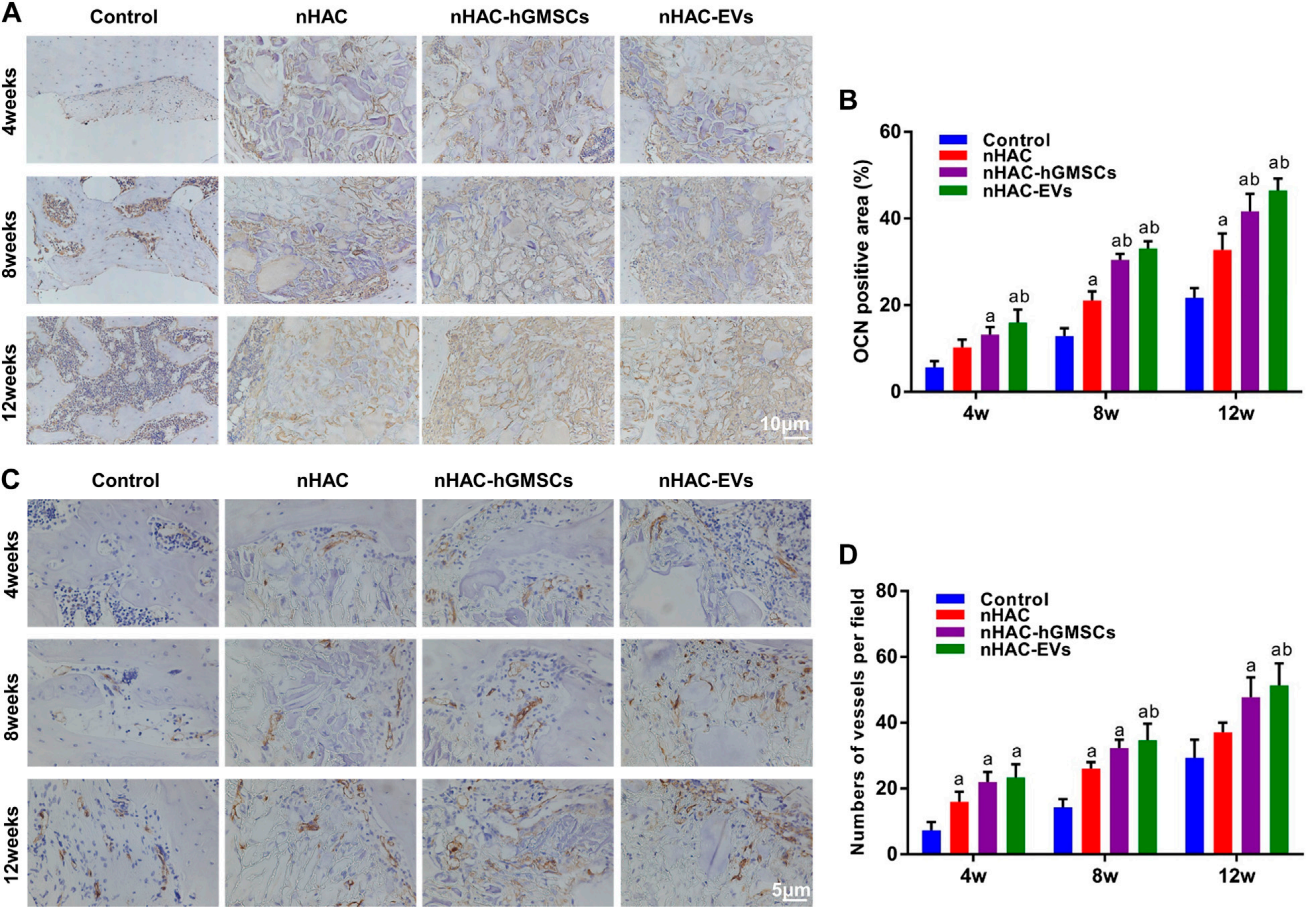
Immunohistochemical staining results of bone regeneration **(A)** Representative images of OCN staining **(B)** Quantitative analysis of OCN expression **(C)** Representative images of CD34 staining **(D)** Quantitative analysis of CD34 expression. a, *p* < 0.05 compared with the control group; b, *p* < 0.05 compared with the nHAC group.

As an important indicator for evaluating osteogenic ability, OCN-positive areas can be stained dark brown by immunohistochemical staining. IHC staining indicated that the expression levels of OCN in groups implanted with nHAC at each time point were higher than those in the control group. Moreover, the nHAC/hGMSCs group and nHAC/EVs group had the highest OCN expression with no significant difference (Figures 9A, B). Additionally, more angiogenesis-related protein CD34 was observed in the nHAC/hGMSCs group and nHAC/EVs group than in the other two groups, suggesting that more new blood vessels were formed in the nHAC/hGMSCs group and nHAC/EVs group (Figures 9C, D).

## 4 Discussion

In general, bone tissue shows good tissue repair function after trauma, but the repair of large-scale bone defects is still a difficult problem in clinical therapy. Bone regeneration is a complex process involving many aspects, such as angiogenesis, osteogenesis, and anti-inflammation (Dimitriou, et al., 2011). Although MSC transplantation therapy has shown good application effects for the repair of bone defects, recent studies have shown that its specific mechanism is mainly accomplished by paracrine effects of MSCs (Rani, et al., 2015; Reis, et al., 2018). As critical paracrine factors secreted by cells, EVs can mediate intercellular communication by delivering proteins, mRNAs, miRNAs and other substances to recipient cells, thereby regulating the biological functions of target cells (Hu, et al., 2021; Tarasov, et al., 2021).

hGMSCs are a kind of MSC with multiple differentiation potential and strong self-renewal ability isolated from the human gingival lamina propria (Fawzy El-Sayed and Dörfer, 2016). The special tissue living environment of the gingiva also makes hGMSCs different from other MSCs. Compared with other MSCs, hGMSCs are easy to obtain, rich in sources, and have good biological properties, showing good application prospects in cell therapy and regenerative medicine (Fawzy El-Sayed and Dörfer, 2016). Several *in vivo* studies have shown that hGMSCs can promote bone regeneration (Xu, et al., 2014; Al-Qadhi, et al., 2020; Hasani-Sadrabadi, et al., 2020). Xu et al. (Xu, et al., 2014) found that hGMSCs could promote the repair of mandibular defects in mice by intravenous injection of hGMSCs applied to the defect. In addition, Al-Qadhi et al. (Al-Qadhi, et al., 2020) indicated that hGMSCs had osteogenic ability similar to that of BMSCs in a tibial defect animal model. However, few studies have reported on bone tissue engineering with hGMSC-derived EVs. A study performed by Jiang et al. (2020) demonstrated that hGMSC-derived EVs could promote the migration and osteogenic differentiation of preosteoblasts. However, the effect of hGMSC-derived EVs on bone defect repair *in vivo* has not been reported. Therefore, the study was conducted *in vitro* and *in vivo* to deeply explore the bone repair effect of hGMSC-derived EVs.

With the deepening of research, it was found that the osteogenic effect of MSC-derived EVs without osteogenic induction is not obvious (Zhang, et al., 2019). Moreover, Liu et al. (Liu, et al., 2020) indicated that the osteogenic differentiation capacity of MSCs could be enhanced by osteogenic induction and that the enhancement effect was related to the time of osteogenic induction of MSCs. This study further showed that the osteogenic effect of EVs after 3 days and 14 days of induction was better (Liu, et al., 2020). However, the stemness and paracrine capacity of MSCs were reduced as the induction time increased (Yeo, et al., 2013). Therefore, in this study, we chose EVs derived from hGMSCs after 3 days of osteogenic induction.

Bone regeneration involves the participation of a variety of cells, and bone-related cells and blood vessel-related cells play an important role (Dimitriou, et al., 2011; Sun, et al., 2022). BMSCs are adult stem cells present in the bone marrow stroma that are activated and mobilized upon injury and serve as the main repair cell type in bone regeneration (Deschaseaux, et al., 2009).

Endothelial cells (ECs) are the first cells to enter the bone marrow after bone tissue injury and coordinate tissue development, maintenance, and regeneration by secreting beneficial vascular secretory signals (Kenswil, et al., 2021). When bone defects occur, BMSCs and ECs can synergistically regulate the bone microenvironment of the defect site and promote bone regeneration by accelerating angiogenesis (Cheng, et al., 2022). Therefore, in this study, we selected rat bone marrow mesenchymal stem cells (rBMSCs) and human umbilical vein endothelial cells (HUVECs) to evaluate the effects of hGMSC-derived EVs on the osteogenic ability of osteoblasts and the angiogenic capability of endothelial cells *in vitro*.

In this study, we found that hGMSC-derived EVs could promote osteogenic differentiation and upregulate the expression of ALP, OCN and RUNX2 osteogenic genes and proteins in rBMSCs. To evaluate the effect of different concentrations of EVs on the osteogenic ability of rBMSCs, we treated rBMSCs with 25 μg/ml, 50 μg/ml and 100 μg/ml EVs. The results showed that 50 μg/ml EVs had the strongest osteogenic ability. In addition, we also demonstrated that hGMSC-derived EVs could enhance the angiogenic ability of HUVECs *in vitro* such that the higher the EVs concentration was, the better the enhancement effect. MSC-derived EVs could promote the osteogenesis of BMSCs and the angiogenesis of HUVECs, which may contribute to the repair of bone defects. Wu et al. (Wu, et al., 2019) found that EVs derived from stem cells from human exfoliated deciduous teeth could enhance the repair of alveolar bone defects through the regulation of osteogenesis of BMSCs and angiogenesis of HUVECs. Moreover, Zhang et al. (Zhang, et al., 2020) also found that BMSC-derived EVs could accelerate fracture healing of non-union through the promotion of osteogenesis and angiogenesis. In this study, hGMSC-derived EVs enhanced the osteogenic ability of rBMSCs and the angiogenic capability of HUVECs. Therefore, we speculated that hGMSC-derived EVs could be an effective approach for bone regeneration *in vivo*.

In animal models of femoral defects, nanohydroxyapatite/collagen was selected as scaffolding material to carry EVs to the site of defects to verify the repair effect of hGMSC-derived EVs on bone defects. In addition, we set up an nHAC/hGMSC group to better evaluate the role of hGMSC-derived EVs. Our data showed that nHAC materials were biocompatible and could be used as application vectors for cells and EVs. At 4, 8 and 12 weeks postsurgery, the bone repair effect of the nHAC/hGMSCs group and the nHAC/EVs group was better than that of the nHAC group and the control group, while the bone repair effect of the nHAC/hGMSCs group and the nHAC/EVs group was not significantly different. Moreover, HE staining, Masson staining, Goldner staining, and immunohistochemical staining for OCN and CD34 showed that more new bone and new blood vessels were produced after treatment with hGMSC-derived EVs, with effects comparable with those of transplanted hGMSCs. In bone tissue engineering, the combination of bone repair materials and bioactive molecules that induce osteogenesis could enhance the function of biomaterials and promote the aggregation and differentiation of osteoblasts, thereby accelerating the repair and regeneration of bone defects (Zuo, et al., 2022). At present, nHAC materials have been widely used in the repair of bone defects, and it is a feasible application strategy to combine them with nanoactive molecular EVs that have good bone induction ability and proangiogenic ability. In this study, hGMSC-derived EVs were able to stimulate osteogenesis of rBMSCs and angiogenesis of HUVECs *in vitro*. In addition, OCN and CD34 were highly expressed in the bone defect areas of the EVs treatment group. Therefore, hGMSC-derived EVs may promote osteogenesis and angiogenesis in bone defect areas by influencing the biological function of endogenous cells, such as BMSCs and ECs. Our findings suggested that the combination of hGMSC-derived EVs with nHAC scaffolds was a reliable method for bone defect repair.

However, this study has some limitations. First, this study did not explore in depth the possible mechanisms of hGMSC-derived EVs in osteogenic differentiation and bone repair at the molecular level. Second, bone regeneration is achieved through bone formation and bone resorption with the participation of osteoblasts and osteoclasts. This study explored the effect of hGMSC-derived EVs on the osteogenic differentiation of osteoblasts, but there is a lack of studies on the biological characteristics of osteoclasts. Finally, in this study, the femoral defect of ordinary rats was selected as the model, and whether it is suitable for femoral defects in osteoporotic rats is not known.

## 5 Conclusion

In this study, we successfully extracted EVs derived from hGMSCs and combined the EVs with a nanohydroxyapatite/collagen scaffold for bone defect repair. Our results demonstrated that the combination of hGMSC-derived EVs and nHAC could significantly promote bone regeneration by advancing osteogenesis and angiogenesis. Therefore, this strategy could serve as a clinical therapy for bone regeneration.

## Data Availability

The original contributions presented in the study are included in the article/supplementary material, further inquiries can be directed to the corresponding authors.
